# Cariprazine alleviates core behavioral deficits in the prenatal valproic acid exposure model of autism spectrum disorder

**DOI:** 10.1007/s00213-021-05851-6

**Published:** 2021-07-15

**Authors:** Viktor Román, Nika Adham, Andrew G. Foley, Lynsey Hanratty, Bence Farkas, Balázs Lendvai, Béla Kiss

**Affiliations:** 1 Gedeon Richter Plc, Budapest, Hungary; 2 AbbVie, Madison, NJ USA; 3 Berand Neuropharmacology Limited, NovaUCD, Belfield Innovation Park, University College Dublin, Dublin, Ireland

**Keywords:** Autism, Cariprazine, Risperidone, Aripiprazole, Core symptom, Social behavior, Repetitive behavior, Hyperactivity, Pharmacotherapy, Neurodevelopmental

## Abstract

**Rationale:**

Autism spectrum disorder (ASD) is a neurodevelopmental condition characterized by deficits in social communication and interaction and restricted, repetitive behaviors. The unmet medical need in ASD is considerable since there is no approved pharmacotherapy for the treatment of these deficits in social communication, interaction, and behavior. Cariprazine, a dopamine D_3_-preferring D_3_/D_2_ receptor partial agonist, is already approved for the treatment of schizophrenia and bipolar I disorder in adults; investigation in patients with ASD is warranted.

**Objectives:**

The aim of this study was to investigate the effects of cariprazine, compared with risperidone and aripiprazole, in the rat prenatal valporic acid (VPA) exposure model on behavioral endpoints representing the core and associated symptoms of ASD.

**Methods:**

To induce the ASD model, time-mated Wistar rat dams were treated with VPA during pregnancy. Male offspring were assigned to groups and studied in a behavioral test battery at different ages, employing social play, open field, social approach-avoidance, and social recognition memory tests. Animals were dosed orally, once a day for 8 days, with test compounds (cariprazine, risperidone, aripiprazole) or vehicle before behavioral assessment.

**Results:**

Cariprazine showed dose-dependent efficacy on all behavioral endpoints. In the social play paradigm, only cariprazine was effective. On the remaining behavioral endpoints, including the reversal of hyperactivity, risperidone and aripiprazole displayed similar efficacy to cariprazine.

**Conclusions:**

In the present study, cariprazine effectively reversed core behavioral deficits and hyperactivity present in juvenile and young adult autistic-like rats. These findings indicate that cariprazine may be useful in the treatment of ASD symptoms.

## Introduction

Autism spectrum disorder (ASD) is a lifelong neurodevelopmental condition characterized by core symptoms of persistent deficits in social communication and social interaction and the presence of restricted, repetitive patterns of behaviors, interests, or activities. ASD is associated with considerable prevalence and burden to the individual, their family, and society (American Psychiatric Association 2013; Lord et al. 2020). Currently, the impaired social relatedness of ASD is managed to a limited extent by behavioral and educational interventions, and despite the high unmet medical need, there is no approved pharmacotherapy for this disorder. Two medications with dopamine D_2_ receptor antagonist or partial agonist properties, risperidone and aripiprazole, respectively, have been approved and are prescribed for the treatment of irritability associated with ASD in the USA and Japan (McCracken et al. 2002; Owen et al. 2009). However, the usefulness of these drugs or compounds of similar pharmacological profile for the treatment of the core symptoms of ASD has not been established. Several studies have investigated the potential efficacy of various psychoactive drugs to improve ASD symptoms, but these studies yielded mixed results (LeClerc and Easley 2015; Posey et al. 2008). The potential side effects of antipsychotics in neurodevelopmental disorders have been reviewed by Lamy and Erickson (2018) and Iasevoli et al. (2020), which concluded that side effects (e.g., weight gain, elevated prolactin levels, extrapyramidal symptoms, somnolence, sedation) may be milder in the younger population (e.g., less than 18 years of age) and less frequent due to lower doses; however, some subgroups may show hypersensitivity to these medications. The clinical results with cariprazine in adults show a beneficial safety profile concerning prolactin levels, sedation, electrocardiogram parameters, and metabolic parameters such as weight gain (Nasrallah et al. 2017). Although only limited data is available with cariprazine in the adolescent population, one study in patients with schizophrenia who were 13 to 17 years of age indicated that cariprazine was generally safe and well tolerated (Szatmári et al. 2020). In this study, adverse events were comparable to the adult population, except for insomnia, which appeared less frequently in adolescents. Further, laboratory parameters, vital sign values, and electroencephalogram parameters were comparable to previously published data in the adult population.

Cariprazine (Vraylar® in the USA and Reagila® in the EU) is a novel dopamine D_3_-preferring D_3_/D_2_ receptor and serotonin 5-HT_1A_ receptor partial agonist (Kiss et al. 2010) that has been approved to treat schizophrenia (USA and Europe) and manic/mixed and depressive episodes associated with bipolar I disorder (USA). Although cariprazine has clearly demonstrated antipsychotic, antidepressant, and antimanic efficacy in multiple clinical studies (Durgam et al. 2015a, b; Németh et al. 2017, Yatham et al. 2020), its potential efficacy in alleviating ASD symptoms has not been investigated. A recently published clinical study based on retrospective chart reviews found improvements after cariprazine treatment in aggression, self-injurious behavior, and impulsivity in patients with ASD, but there has not been any prospective, placebo-controlled clinical trial of cariprazine in ASD (Cohen and Pella 2019).

With the lack of clinical trial data, preclinical studies could assess the potential of cariprazine for the treatment of ASD. There is some indirect preclinical evidence already available, such as neurophysiological findings. Specifically, an excitatory and inhibitory imbalance in the brain is a key hypothesis of ASD neurophysiology, which is associated with abnormal gamma-band oscillations and suggested to be linked with perceptual and cognitive functions that are compromised in autism (David et al. 2016; Peiker et al. 2015; Rojas and Wilson 2014). Findings of an ex vivo study by Meier et al. (2019) demonstrate a stabilizing role of cariprazine on gamma oscillations, presumably due to its partial agonist properties on dopamine D_3_ receptors. While changes in brain oscillation are not exclusively specific to ASD, more direct evidence can be gained from dedicated preclinical disease models of ASD such as behavioral investigation after prenatal exposure to valproic acid (VPA) in rodents. The VPA model has high translational value as its induction, and manifestations on multiple levels of organization show similarities to the human ASD condition (Mabunga et al. 2015; Nicolini and Fahnestock 2018). Fetal exposure to VPA during pregnancy is associated with an increased risk of children being born with birth defects or developing symptoms of autism in childhood (Lord et al. 2020). Based on the above, the aim of the present study was to investigate cariprazine’s effect in the prenatal VPA autism model using rats by assessing various behavioral endpoints focusing on impaired social interaction and repetitive behaviors that pertain to ASD. Risperidone and aripiprazole were also investigated as comparators.

## Materials and methods

### Animals

In order to induce an autistic-like condition in the offspring, time-mated female Wistar rats (Harlan, UK) were treated with VPA (Sigma Aldrich, batch number: MKCB2699V) intraperitoneally (i.p.) at a single dose of 600 mg/kg on gestational day 12.5; a saline-injected (i.p.) group was included as a vehicle control. Upon weaning, all juvenile animals were examined for characteristic “tail kinks” to ensure fetal exposure to VPA. Offspring were housed according to standard laboratory conditions until commencement of the experimental procedure. Due to the overrepresentation of males in ASD (Loomes et al. 2017), only male offspring were randomly assigned to experimental groups (*n* = 8/group) and tested at juvenile age (postnatal days 30 and 31) and as young adults (postnatal days 59 and 60). Group housing assignments were randomized across litters. Also, animals were randomized when assigning them to treatment groups. Treatment groups comprised the same animals (first tested as a juvenile, then as an adult). Prior to behavioral assessment, animals were transferred to experimental holding rooms where they were housed in groups of 4 on a standard 12-h light–dark cycle with lights on at 07:30 and ambient temperature at 22–24 °C. Food and water were available ad libitum. All procedures were carried out by individuals retaining the appropriate license from the Irish Government Health Products Regulatory Authority; furthermore, the study protocol was approved by the Animal Research Ethics Committee of University College Dublin, Ireland. All efforts were made to reduce the number of experimental animals involved as well as their suffering. Experiments were carried out in strict compliance with the European Directive 2010/63/EU regarding the care and use of laboratory animals for experimental procedures.

### Test compounds

Cariprazine hydrochloride (synthesized at Gedeon Richter Plc., batch number L78005N), risperidone (Tocris, batch numbers 1B/154,067 and 1B/218,115), and aripiprazole hydrochloride (synthesized at Gedeon Richter Plc., batch number 0003A96) stocks were stored at room temperature or 4 °C. Fresh solutions were prepared daily in 5% Tween 80 suspended in distilled water. Test compounds were administered by oral gavage daily for 7 days prior to (postnatal days 23–29 and 52–58) experimental testing. Dosing continued on the days of behavioral assays (postnatal days 30–31 and 59–60) when test compounds were administered 1 h prior to testing. Treatment groups were vehicle (prenatal saline and study drugs’ vehicle [5% Tween 80 in distilled water]; VEH), a VPA-exposure control (prenatal valproate and study drugs’ vehicle; VPA), cariprazine (prenatal valproate and 0.003, 0.01, 0.03, and 0.1 mg/kg cariprazine; CAR), risperidone (prenatal valproate and 0.1 mg/kg risperidone; RIS), and aripiprazole (prenatal valproate and 1 mg/kg aripiprazole; ARI). Drug dosages were chosen based on findings with cariprazine, aripiprazole, and risperidone in behavioral studies (Gyertyán et al. 2011; Neill et al. 2016; Watson et al. 2016) and were carefully selected to avoid the occurrence of any locomotor side effects but at levels efficacy may be expected. All doses presented in the paper refer to base compounds.

### Behavioral assays

Social play was assessed on postnatal day 30. The test was carried out under low light in a novel, high-walled, open-field test arena (40 × 40 cm), wherein paired animals from the same treatment group, but different cages, were examined. First, animals were allowed to individually explore the arena for a 10-min acclimatization trial (postnatal day 29); 24 h later, they were evaluated for social play for 15 min (postnatal day 30). Play activity was measured by observing pinning and mounting behavior (latency to, total duration, and frequency) and duration of general social behavior (following, chasing, sniffing, licking, or social grooming of test partner). Scoring was conducted in a blinded fashion by manual video analysis employing Ethovision XT (Noldus, UK) software linked to an overhead video camera; values were expressed as mean ± standard error of the mean (SEM). Each data point represented one pair of rats.

Locomotor activity and exploratory behavior were assessed in an open-field arena on postnatal day 31. Animals were placed in a black Perspex arena (64 × 64 cm) with 30 cm tall side walls, under low light conditions, and monitored via remote video capture for spontaneous activity. Videos were analyzed using Ethovision XT image software (Noldus, UK) to determine total distance traveled and number of center zone crossings. Frequency of circling behavior (number of 360° turns) and grooming events were determined manually by a blinded investigator. All measures were expressed as mean ± SEM.

**Fig. 1:**
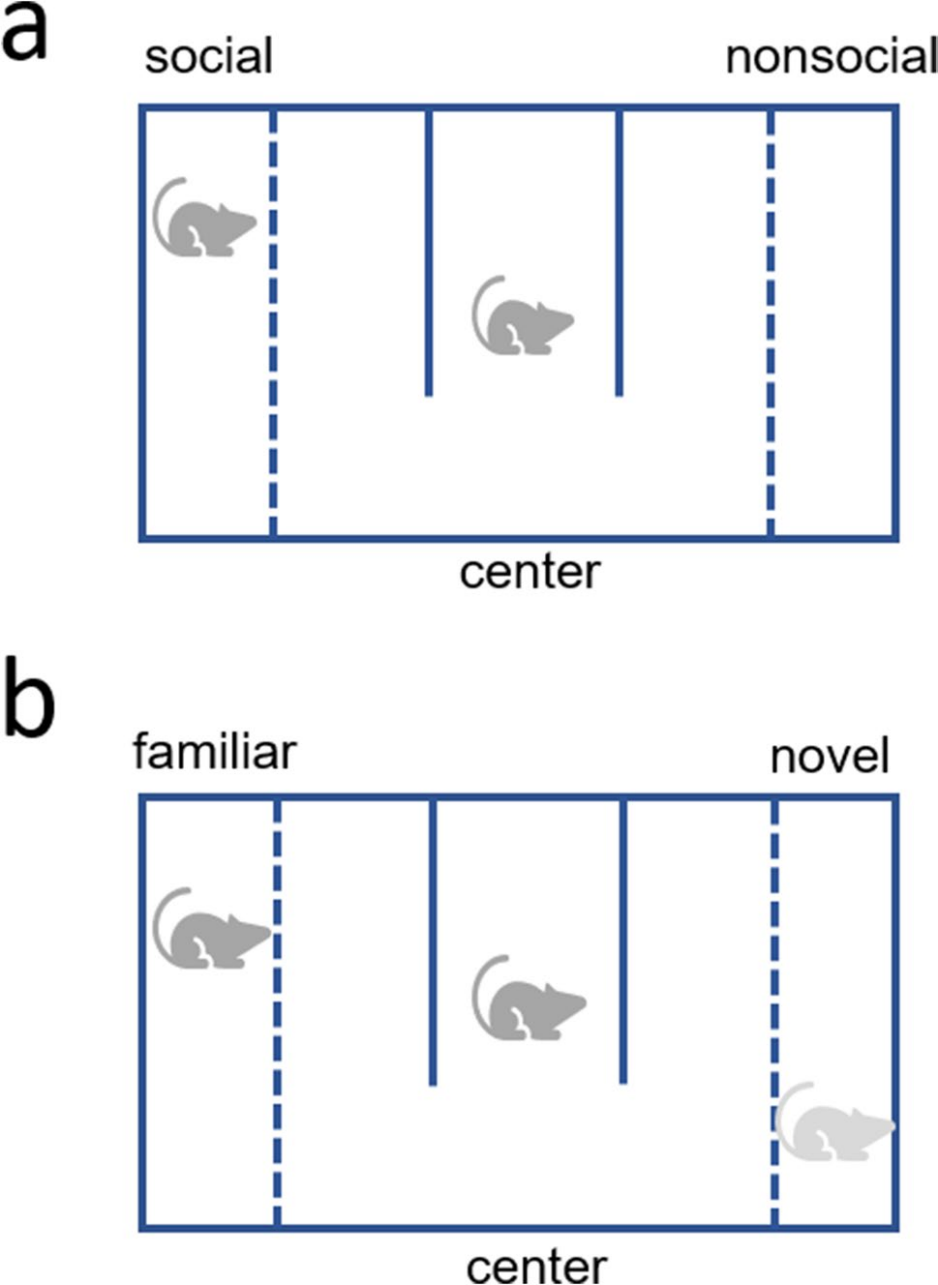
Schematic of (**a**) the social approach-avoidance test in the 3-chamber apparatus and (**b**) the social recognition memory test in the 3-chamber apparatus

Social approach-avoidance was assessed on postnatal day 59 in a 3-chamber apparatus (Foley et al. 2012). The 3-chamber apparatus was a specially constructed black Perspex rectangular box (65 cm long × 30 cm wide × 35 cm high) with 2 identical chambers (10 cm long × 30 cm wide × 35 cm high) separated from a central area, which was further divided by 2 black Perspex baffles 10 cm away from the outer wall. In the side chambers, 2 transparent Perspex sheets (1 on each side) with multiple, small, evenly-placed holes over their surface separated holding compartments to enclose stimulus animals (Fig. 1a). Stimulus animals were rats similar to experimental animals with respect to strain, gender, and size and randomly assigned to either chamber during each test. During a 5-min acclimatization trial, test animals were allowed to first freely explore the central area; afterwards, the stimulus animal was placed in one of the side chambers, and the test animal was returned to the test apparatus (starting in the center chamber) for the 5-min social approach-avoidance test trial. Since the Perspex baffles were immobile and present throughout testing, this set up did not allow for the separation of social interest from the effect of novelty. The time spent in each chamber (social, center, or nonsocial) and time spent displaying active social behavior with the stimulus animal at the separation wall (active social behavior was defined as the following: being in the proximity of the stimulus animal at the baffle, orienting, nose poking, sniffing, licking, chewing, and paw reaching towards the stimulus animal) was monitored using an overhead video camera; scoring was also conducted by a blind investigator via manual video analysis using Ethovision XT (Noldus, UK). All measures were expressed as mean ± SEM.

Social recognition memory was assessed 1 day later, on postnatal day 60. Using the same apparatus as the social approach-avoidance test (postnatal day 59), the test animal was returned to the central area, except in the presence of a familiar animal (stimulus animal from the social approach-avoidance test on the previous day) in one chamber and a novel stimulus animal in the other chamber, for a 5-min “choice” test trial. The time spent in each chamber (familiar, center, or novel; Fig. 1b) is calculated and expressed as mean ± SEM; scoring is also conducted by a blind investigator via manual video analysis using Ethovision XT (Noldus, UK). All measures were expressed as mean ± SEM.

### Statistical analysis

For the social play and the open-field test, one-way ANOVA (ordinary or Brown-Forsythe ANOVA, if Bartlett test indicated there were significant differences in standard deviation between groups) was applied to find drug treatment effects, followed by post-hoc Dunnett’s multiple comparisons versus VPA.

For the social approach-avoidance and social recognition memory assays, time spent in chambers was analyzed by two-way repeated measures ANOVA, with chamber and treatment as factors, followed by Tukey’s multiple comparisons test to make between (†) and within (*) chamber comparisons. With respect to active social time in the social approach-avoidance assay, one-way ANOVA was applied, followed by post-hoc Dunnett’s multiple comparisons versus VPA. With respect to active social time in the social recognition memory assay, two-way ANOVA was used, with chamber and treatment as factors, followed by Tukey’s multiple comparisons test for between (†) and within (*) chamber comparisons.

Significance was set at *p* < 0.05 for all behavioral tests.

## Results

### Social play

**Fig. 2:**
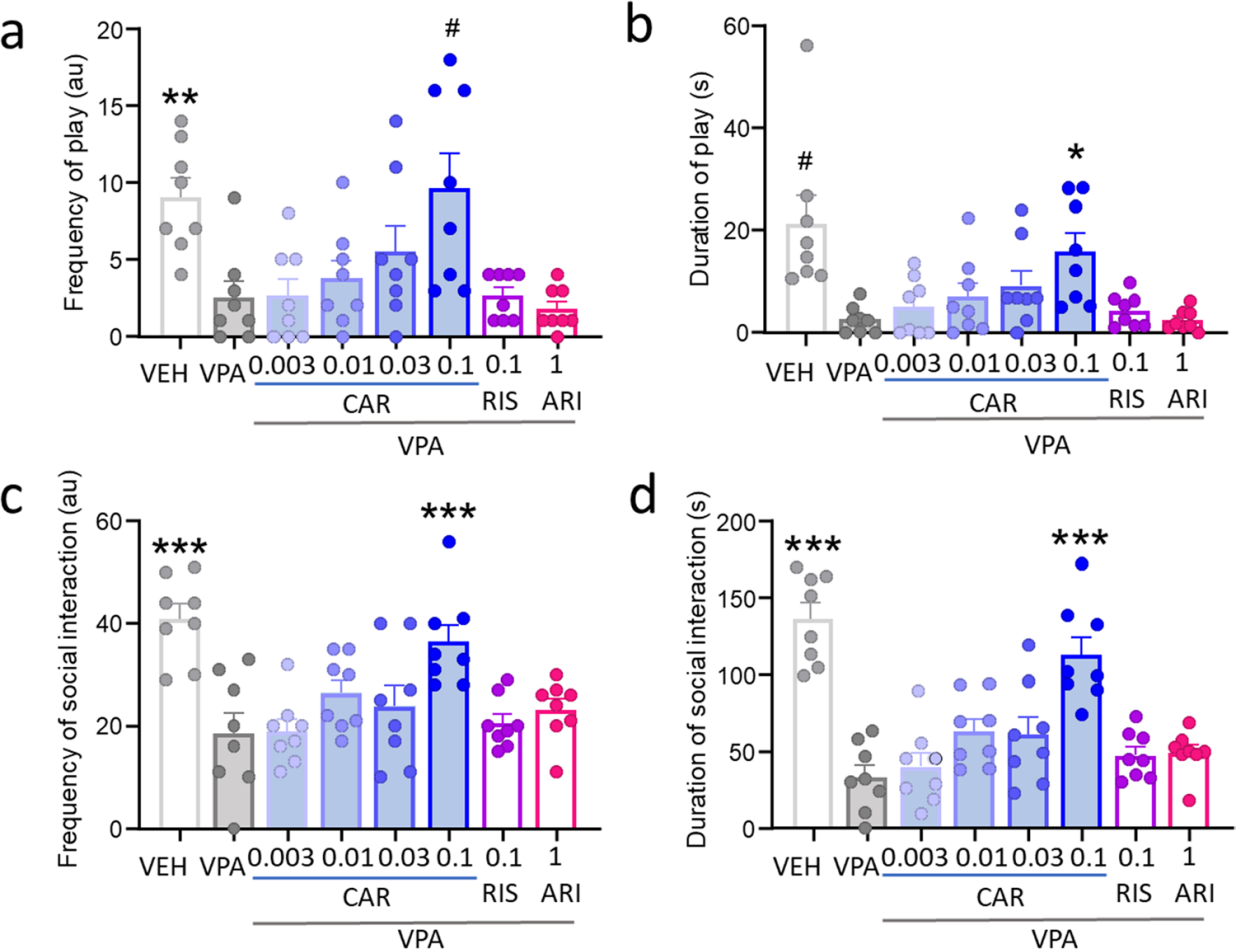
Cariprazine reversed VPA-induced deficits in (**a**), (**b**) juvenile social play and (**c**), (**d**) reciprocal social interaction on postnatal day 30. Risperidone and aripiprazole did not have any effects on any endpoints in this assay. #, *, **, *** *p*<0.1, 0.05, 0.01, 0.001 vs VPA (Dunnett’s multiple comparisons)

Juvenile rats are examined for social play behavior, such as short bouts of mounting and wrestling behavior, and reciprocal social interaction on postnatal day 30 (Fig. 2). The prenatally VPA-exposed control group exhibits a significant decrease in the number (*p* = 0.0092; Fig. 2a) and a near significant reduction in the duration (#*p* = 0.0590; Fig. 2b) of play activities. Prenatal VPA exposure also significantly reduces the frequency (*p* < 0.0001; Fig. 2c) and duration (*p* < 0.0001; Fig. 2d) of social interactions. Drug treatment had a significant effect on frequency (*F*(7.000, 32.04) = 5.68, *p* = 0.0003) and duration (*F*(7.000, 25.25) = 5.788, *p* = 0.0004) of social play in prenatally VPA-exposed rats. Cariprazine treatment reversed the VPA-induced deficits of social play in a dose-dependent manner (Fig. 2); effects on frequency almost reached significance at the highest dose of 0.1 mg/kg (#*p* = 0.0935 vs. VPA; Fig. 2a). Duration of play was significantly increased by cariprazine at 0.1 mg/kg (*p* = 0.0398 vs VPA). Social interaction was dose-dependently improved by cariprazine treatment with significant effects at the dose of 0.1 mg/kg both for number (*p* < 0.0001 vs VPA) and duration of social interactions (*p* < 0.0001 vs VPA). Risperidone and aripiprazole did not affect any behavioral elements in this assay (Fig. 2).

### Open-field locomotor activity

**Fig. 3:**
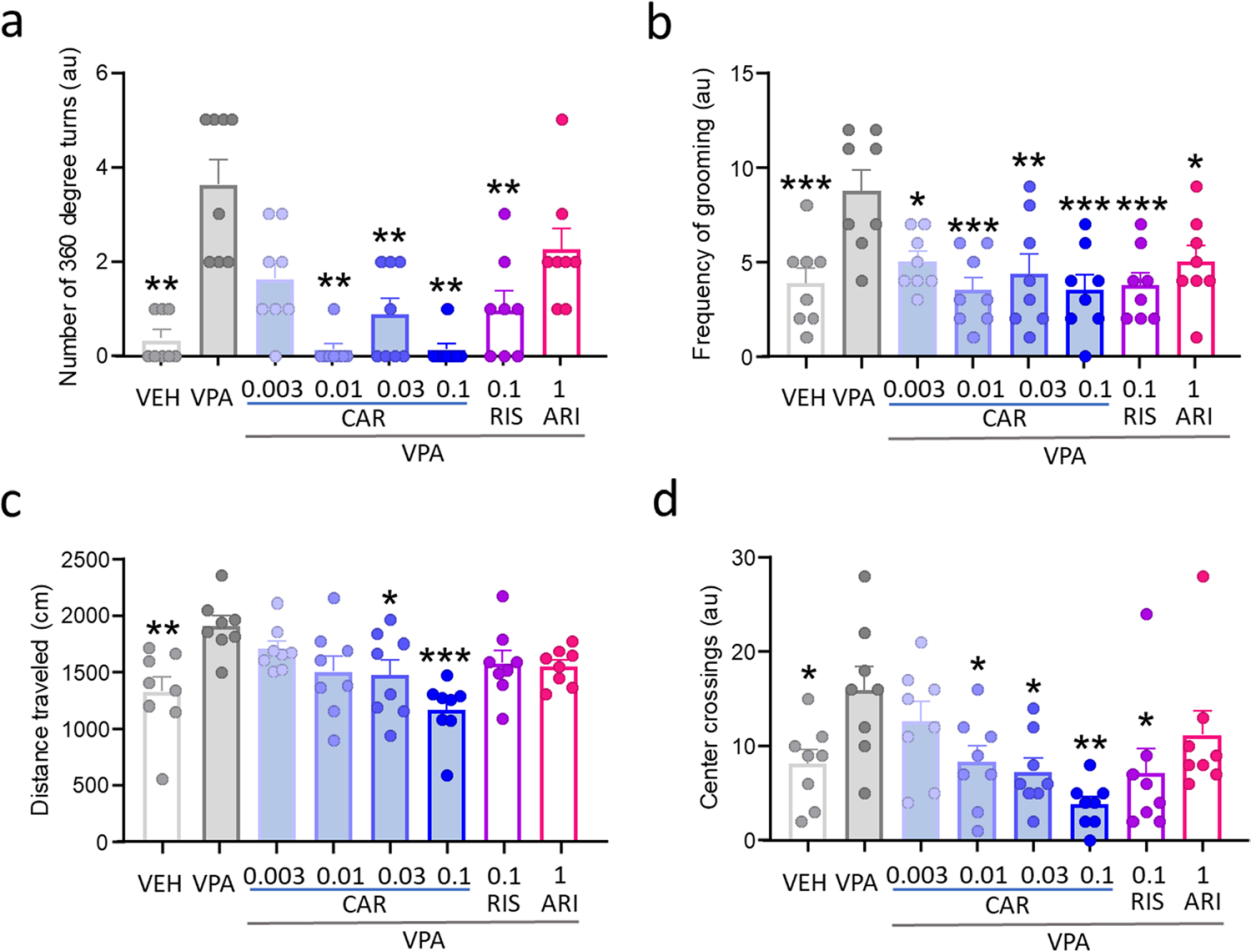
Cariprazine reduced VPA-induced stereotypical behavior (**a** circling; **b** grooming) and hyperactivity (**c** distance travelled; **d** center crossings) in the open-field assay on postnatal day 31. Risperidone and aripiprazole also reduced excessive repetitive behavior, and risperidone reduced center crossings. *, **, *** *p*<0.05, 0.01, 0.001 vs VPA (Dunnett’s multiple comparisons)

Hyperactivity (distance traveled and center crossings) and stereotypical behavior (circling and excessive grooming) are examined on postnatal day 31 (Fig. 3). The VPA-exposed control group exhibits excessive circling (*p* = 0.0017; Fig. 3a), grooming (*p* = 0.0008; Fig. 3b), general locomotor activity (*p* = 0.0019; Fig. 3c), and center crossings (*p* = 0.0431; Fig. 3d) compared with the vehicle-treated group. Drug treatment had a significant effect on the number of 360° turns (*F*(7.000, 35.95) = 12.24, *p* < 0.0001; Fig. 3a) and frequency of grooming (*F*(7,56) = 4.37, *p* = 0.0006; Fig. 3b). Cariprazine significantly reduced VPA-induced circling behavior in the dose range of 0.01 to 0.1 mg/kg (*p* = 0.0013, 0.0066, and 0.0013 for 0.01, 0.03, and 0.1 mg/kg vs VPA, respectively). Similarly, cariprazine significantly decreased VPA-induced excessive grooming at all doses (*p* = 0.0138, 0.0003, 0.0029, and 0.0003 for CAR 0.003, 0.01, 0.03, and 0.1 mg/kg vs VPA, respectively). Risperidone reduced excessive circling (*p* = 0.0096 vs VPA) and grooming (*p* = 0.0006 vs VPA) induced by prenatal VPA exposure. Aripiprazole did not significantly decrease circling, but it significantly reduced grooming (*p* = 0.0138 vs VPA). Drug treatment significantly affected distance traveled (*F*(7,56) = 4.449, *p* = 0.0005, Fig. 3c) and number of center crossings (*F*(7,56) = 3.61, *p* = 0.0028; Fig. 3d). Cariprazine dose-dependently reduced VPA-induced locomotor hyperactivity, reaching significance at the dose of 0.03 mg/kg (*p* = 0.0325 vs VPA) and 0.1 mg/kg (*p* < 0.0001 vs VPA). Cariprazine significantly diminished the number of center crossings in the dose range of 0.01 to 0.1 mg/kg (*p* = 0.0481, 0.0192, and 0.0005 for 0.01, 0.03, and 0.1 mg/kg vs VPA, respectively). Risperidone and aripiprazole did not significantly decrease distance traveled in this assay. Risperidone significantly reduced the number of center crossings (*p* = 0.0170 vs VPA), while aripiprazole treatment did not significantly affect this parameter.

### Social approach-avoidance

**Fig. 4:**
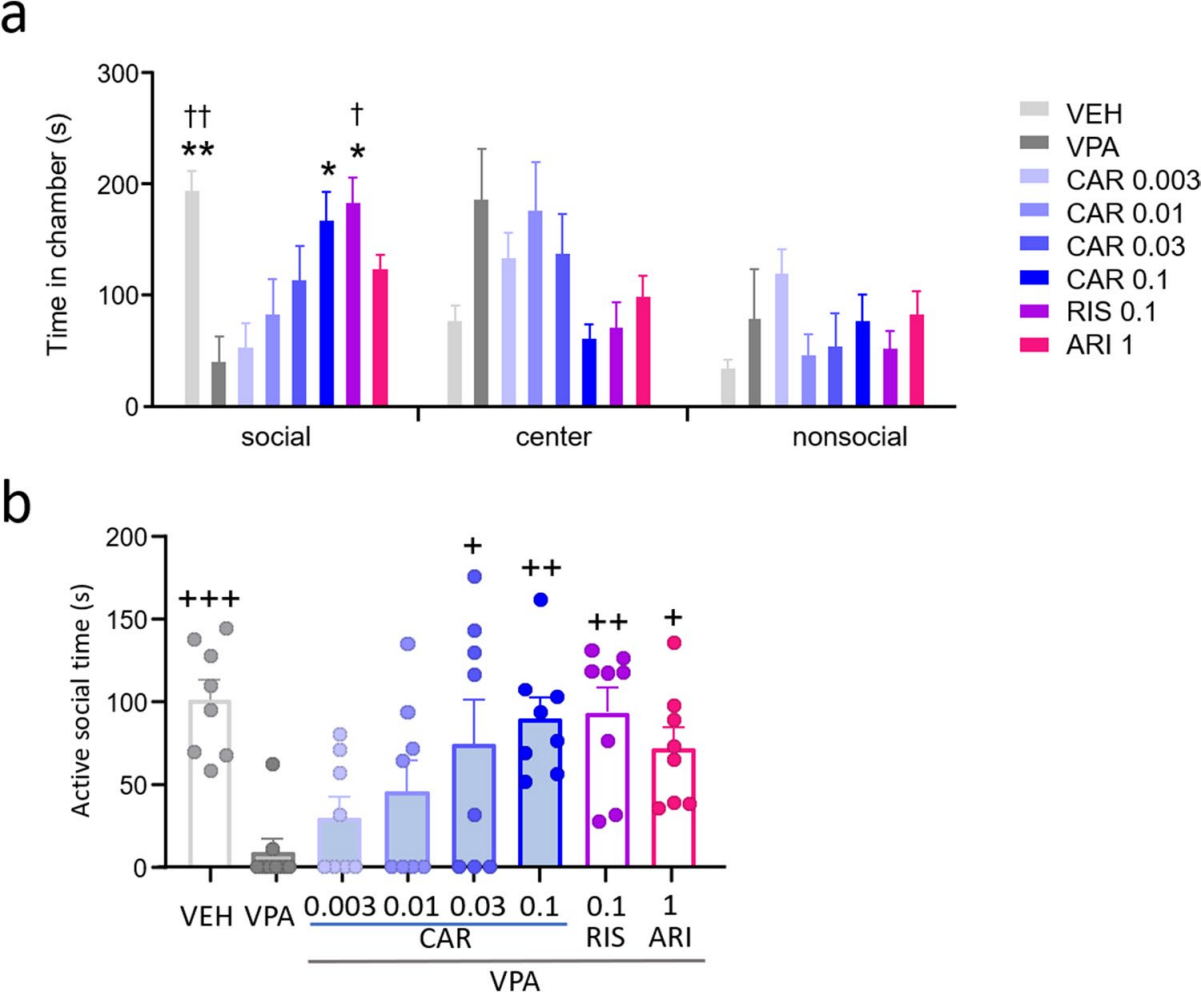
Cariprazine, risperidone, and aripiprazole reversed VPA-induced social deficits in the social approach- avoidance test on postnatal day 59. (**a**) Cariprazine and risperidone increased time spent in the social chamber. (**b**) Cariprazine dose-dependently restored time spent with active social investigation of the stimulus rat; risperidone and aripiprazole also increased social activity. *, ** *p*<0.05, 0.01 vs VPA (Tukey’s multiple comparisons); †, †† *p*<0.05, 0.01 vs nonsocial (Tukey’s multiple comparisons); +, ++, +++ *p*<0.05, 0.01, 0.001 vs VPA (Dunnett’s multiple comparisons)

Young adult rats are tested for social behavior and social preference in the 3-chamber social approach-avoidance paradigm on postnatal day 59 (Fig. 4). Prenatal VPA exposure significantly reduced the time spent in the social chamber compared with VEH (*p = 0.0045 vs VPA). Vehicle controls significantly preferred the social to the nonsocial chamber (†*p* = 0.0014 vs nonsocial). The VPA-induced reduction in social chamber time was restored by 0.1 mg/kg cariprazine (*p = 0.0497 vs VPA) and risperidone (*p = 0.0143 vs VPA). In case of risperidone, there was a significant preference for the social over the nonsocial chamber (†*p* = 0.0197 vs nonsocial). Prenatal VPA treatment significantly decreased the time spent with active social investigation compared with vehicle controls (*p* = 0.0007 vs VPA; Fig. 4b). There is a significant drug treatment effect (*F*(7,56) = 4.495, *p* = 0.0005; Fig. 4b). Cariprazine dose-dependently reversed the time in social investigation, reaching significance at the dose of 0.03 mg/kg (*p* = 0.0244 vs VPA). Risperidone and aripiprazole also significantly increased social active time (*p* = 0.0020 and *p* = 0.0342 vs VPA, respectively).

### Social recognition memory

**Fig. 5:**
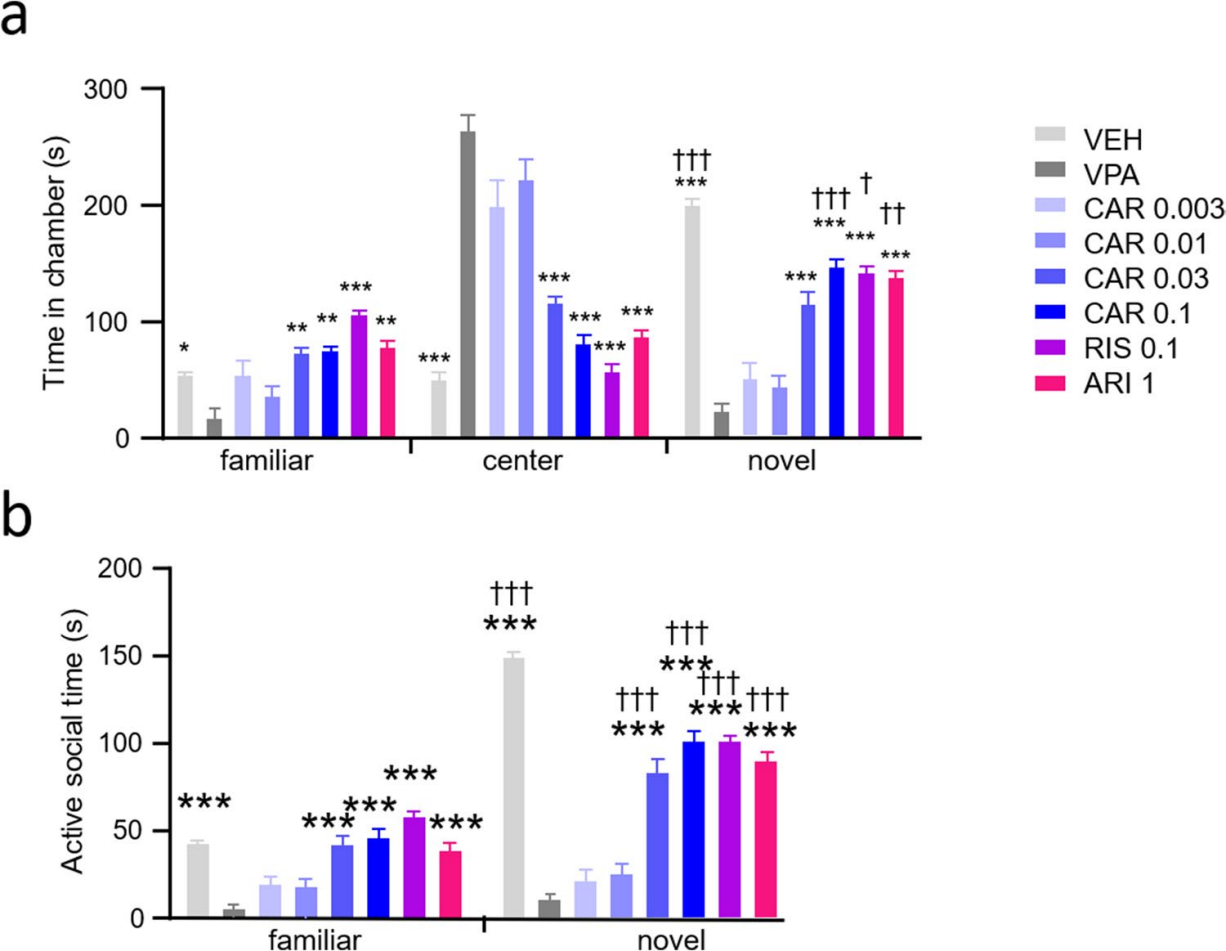
Cariprazine, risperidone and aripiprazole reversed VPA-induced social deficits in the social recognition memory test on postnatal day 60. All three compounds increased (**a**) the time spent in the chamber containing the novel conspecific and (**b**) the time spent with active investigation of the novel stimulus rat. *, **, *** *p*<0.05, 0.01, 0.001 vs VPA (Tukey’s multiple comparisons); †, ††, ††† *p*<0.05, 0.01, 0.001 vs familiar (Tukey’s multiple comparisons)

Young adult rats rested for 24 h after the social approach-avoidance test before being returned to the 3-chamber apparatus for the social memory test on postnatal day 60 (Fig. 5). Prenatal VPA exposure significantly reduced time spent in the chamber containing the novel conspecific (*p < 0.0001) and concomitantly increased time spent in the center chamber (*p < 0.0001) versus VEH. Vehicle controls significantly preferred the chamber with the novel rat compared with the chamber with the familiar stimulus animal (†*p* < 0.0001 vs familiar). The VPA-induced reduction in the time spent in the chamber containing the novel rat was restored by 0.1 mg/kg cariprazine, risperidone, and aripiprazole (†*p* = 0.0008, 0.0161, 0.0041 vs familiar, respectively). Prenatal VPA treatment significantly reduces social investigation directed towards the familiar as well as the novel stimulus rat (*p < 0.0001 vs VPA for both), whereby the social novelty preference present in VEH controls (†*p* < 0.0001 vs familiar) disappeared in the VPA group (Fig. 5b). Treatment with cariprazine (0.03 and 0.1 mg/kg), risperidone, and aripiprazole increased social activity towards the familiar and novel conspecifics (*p < 0.0001 vs VPA for all), which resulted in the restoration of social novelty preference by these compounds and doses (†*p* < 0.0001 vs familiar for all).

## Discussion

This study investigated cariprazine’s potential therapeutic efficacy in ASD using the prenatal VPA-exposure model in rats. The prenatal VPA-exposure model is widely accepted as a model of ASD with high translational value due to its similarities to the human condition with respect to its induction and phenotypic manifestation (Mabunga et al. 2015). Indeed, there is supporting evidence that fetal exposure to VPA during pregnancy is associated with an increased risk of children being born with birth defects or developing symptoms of autism in childhood (Lord et al. 2020). Although there may be only a small number of prenatal VPA-exposure cases in the overall ASD population, the induced excitatory/inhibitory imbalance in this model could be predictive of ASD (Gogolla et al. 2009; Lee et al. 2017; Sohal and Rubenstein 2019). Similar to humans, fetal exposure to VPA in rats or mice may result in ASD-like phenotypic changes ranging from the cellular to behavioral level in the offspring (Nicolini and Fahnestock 2018; Pelsőczi et al. 2020; Spisák et al. 2019). Thus, the VPA-exposure model has the advantage of a solid scientific rationale compared with other models, such as idiopathic models with poor construct validity or genetic models that although may well have a clear basis, yet each represents only a small fraction of the whole ASD spectrum (El-Fishawy and State 2010). Predictive validity of the prenatal VPA-exposure model, like any ASD model, is difficult to assess since there is no approved drug on the market to treat the socio-communicational aspects of ASD.

In this study, cariprazine was effective in the prenatal VPA-exposure model of ASD in rats. Specifically, in juvenile and young adult rats displaying autistic-like symptoms, cariprazine significantly reduced core behavioral deficits and hyperactivity. Moreover, it showed a significant reversal of disrupted social play behavior, a chief indicator of normal development. Social play is the earliest form of social behavior that is not directed towards the mother but towards other conspecifics of the same age (Trezza et al. 2010; Vanderschuren and Trezza 2014). Most young mammals show some form of rough and tumble social play, with certain species, such as rats, demonstrating this behavior more so than mice. In contrast to earlier ASD-related preclinical models where antipsychotic agents were tested in mice, the present study used rats. Compared with mice, rats have a more developed communication system and show a wider set of social behavioral elements with easily identifiable mutual social play behavior (Brust et al. 2015; Homberg et al. 2017). There was a striking difference between cariprazine and aripiprazole or risperidone in the social play test. The finding that cariprazine but not aripiprazole and risperidone were effective in the social play domain is particularly relevant since the ability to participate in social play is a leading sign of healthy development of social communication both in animals and humans. Similar to prenatally VPA-exposed rats that display significantly low levels of rough and tumble play, children with ASD are also less likely to engage in interactions with other children, and their play patterns are more asocial and object-oriented (Jordan 2003). Not only did cariprazine reverse the social play deficits in this behavioral assay, but it also reversed general social behavior in the same young rats, whereas risperidone and aripiprazole were ineffective on this behavioral endpoint.

There are further examples for the differentiated activity of cariprazine versus other atypical antipsychotics on various social behavioral endpoints in other models. For example, in a subchronic phencyclidine (PCP) model, both risperidone and cariprazine reduced social avoidance, while only cariprazine was able to restore following behavior (Neill et al. 2016). Similarly, in a study by Watson et al. (2016), the body sniffing element of social interaction was normalized only by cariprazine and not by aripiprazole in neonatally PCP-treated and socially isolated rats. The explanation for these differences in efficacy may rest in the complex and unique receptor affinity and functional profile of cariprazine relative to the other atypical antipsychotics with special regards to a prominent role of dopamine D_3_ receptors. Cariprazine possesses a unique receptor signature; it shows subnanomolar affinity for rat (and human) dopamine D_3_ and D_2_ receptors with 5- to eightfold D_3_ selectivity over D_2_ receptors and partial agonist activity at these receptors in vitro (Kiss et al. 2010; Tadori et al. 2011a). Further, cariprazine occupies both D_2_ and D_3_ receptors in the rat and human brain in vivo (Girgis et al. 2016; Gyertyán et al. 2011; Kiss et al. 2012). Aripiprazole displays nanomolar affinity for both D_2_ and D_3_ receptors with approximately 3- to fivefold preference for D_2_ versus D_3_ receptors and also behaves as a partial agonist at both receptors in vitro (Kiss et al. 2010; Tadori et al. 2011a). However, aripiprazole, in contrast with cariprazine, shows little, if any, in vivo D_3_ receptor occupancy in either the rat or human brain (Girgis et al. 2017; Gyertyán et al. 2011; Kiss et al. 2012). The above properties may represent further differentiating aspects of the two partial agonists on social play. Risperidone is a D_2_ and D_3_ receptor antagonist/inverse agonist with about 3- to fourfold higher binding affinity for D_2_ than for D_3_ receptors in vitro (Burstein et al. 2005; Hertel et al. 1996; Kiss et al. 2010; Tadori et al. 2011b). It displays only D_2_ but no D_3_ receptor occupancy in the rat (McCormick et al. 2010; Kiss et al. 2012) and human brain in vivo (Graff-Guerrero et al. 2009; Mizrahi et al. 2011). In a recent publication, the dopamine D_3_ receptor preferential antagonist, F17464, seemed to produce some efficacy in a prenatal VPA-exposure model, whereas aripiprazole had very limited efficacy (Cosi et al. 2020).

The dopamine D_3_ receptor is abundantly expressed in the nucleus accumbens (Sokoloff and Le Foll 2017), which is known to be involved in the regulation of social play (Manduca et al. 2016). In this brain area, cariprazine induces the release of various neurotransmitters including dopamine, norepinephrine, serotonin, glycine, and glutamate (Huang et al. 2019) and upregulates D_3_ receptor expression (Choi et al. 2014) following chronic treatment. Moreover, given that cariprazine displays partial agonism at dopamine D_3_ and D_2_ receptors and has high in vivo D_3_ receptor occupancy of both receptors (Gyertyán et al. 2011; Kiss et al. 2012), it is possible that cariprazine indirectly stabilizes GABAergic transmission in autism-relevant brain regions such as the nucleus accumbens or the amygdala (Chen et al. 2006; Diaz et al. 2011). Another indirect means of the prosocial effects of cariprazine may originate from the release of the neurohormone oxytocin that has been shown to be regulated by D_3_ receptors (Uvnäs-Moberg et al. 1995). The notion that the D_3_ receptor most likely plays an important role in the prosocial effects of cariprazine is also supported by the finding that cariprazine’s efficacy in ameliorating social behavior deficits in a schizophrenia model in wild-type mice is absent in mice lacking D_3_ receptors (Zimnisky et al. 2013). Furthermore, other aspects of social behavior, such as huddling in rats as well as social investigation in mice, can be altered by D_3_ receptor ligands (Gendreau et al. 2000; Kagaya et al. 1996; Rodríguez-Arias et al. 1999). Also, the differentiating D_3_ receptor profile of cariprazine may explain its greater potency relative to other atypical antipsychotics in all of these behavioral assays, which is in agreement with earlier results in various behavioral tests including MK-801- and PCP-induced hyperlocomotion or a water labyrinth test (Gyertyán et al. 2011).

Cariprazine, similar to risperidone and aripiprazole, was effective in reducing social deficiencies in the 3-chamber assay and repetitive behaviors (excessive grooming and circling). Additionally, all 3 drugs diminished the hyperactivity (e.g., distance traveled and center crossings) of VPA-treated rats in the open field, which is congruent with the use of risperidone and aripiprazole in children and adolescents with ASD (Lamy and Erickson 2018). However, efficacy of the investigated drugs in the 3-chamber assay is harder to judge from a translational point of view due to absence of any approved medication for the treatment of core symptoms of ASD. With respect to improvements in the socio-communicational domain, results from clinical studies with risperidone and aripiprazole have been mixed. While risperidone resulted in modest improvements of the core symptoms in children with pervasive developmental disorders exhibiting high levels of baseline irritability, aripiprazole did not improve lethargy/social withdrawal in ASD (LeClerc and Easley 2015; Posey et al. 2008). Similar to the clinical data, results of these two compounds in models of ASD (e.g., prenatal VPA in rats or mice, BTBR mice) have also been inconsistent. Regarding treatment duration, acute regimens are usually ineffective (Chadman 2011; Cosi et al. 2020; Gould et al. 2011; Hara et al. 2017), while repeated dosing schemes can deliver efficacy signals (Hara et al. 2017); however, some preclinical findings with risperidone from transgenic models oppose this trend, as acute risperidone produced some effect in Oprm1 KO mice (Becker et al. 2014), while chronic administration in Cntnap2 KO mice did not (Penagarikano et al. 2011). Nevertheless, in the present study, efficacy was demonstrated for all 3 drugs in the 3-chamber assay after repeated administration, which does not contradict the aforementioned preclinical or clinical findings. Although the 3 investigated compounds did not differentiate on the social approach-avoidance and social recognition memory assays, cariprazine did have measurable effects that may warrant clinical trials in the assessment of its efficacy in ASD.

While cariprazine’s prosocial effect in prenatally VPA-exposed rats is a novel finding, there were earlier findings of beneficial effects on general sociability in models of negative symptoms of schizophrenia and psychosis. In studies using acute PCP treatment, cariprazine improved social interaction and social recognition memory deficits in mice (Zimnisky et al. 2013) and rats (Watson et al. 2016). Cariprazine’s general prosocial and, in particular, social recognition memory improving effects may eventually be associated with its known procognitive efficacy (Gyertyán et al. 2011; Neill et al. 2016; Watson et al. 2016). Similarly, improvement of social recognition memory after risperidone treatment in the present study may be attributable to its overall procognitive effect, which has been reported in subjects with schizophrenia after risperidone dosing (Woodward et al. 2005). Procognitive efficacy of these compounds may be regulated via the frontocortical dopamine D_3_ receptors (Kehr et al. 2018; Loiseau and Millan 2009), which has been shown to be instrumental for proper social behavior (Selimbeyoglu et al. 2017; Yizhar et al. 2011). Notwithstanding the fact that the frontal cortex expresses low levels of D_3_ receptors (Meador-Woodruff et al. 1996), there is mounting evidence suggesting that these receptors are important in frontocortical processes (Nakajima et al. 2013). Although the exact mechanism whereby D_3_ receptors influence frontocortical functions is not fully known, a potential means may be the modulation of cortical excitability. Earlier studies implicate modulation of cholinergic and glutamatergic transmission by D_3_ receptor-mediated changes in cortical excitability (Millan et al. 2007; Sokoloff et al. 2013). A more recent compelling explanation is that cariprazine may modulate excitability by influencing Ca_v_3 type calcium channels in pyramidal cells of the prefrontal cortex through D_3_ receptors (Clarkson et al. 2017; Yang et al. 2016).

The inhibition of excessive repetitive and locomotor behavior in the prenatal VPA-exposure model used here was most likely not due to detrimental motor effects because the dose range of cariprazine (0.003–0.1 mg/kg) or the doses of risperidone (0.1 mg/kg) and aripiprazole (1 mg/kg) were well below doses causing motor impairment (Gyertyán et al. 2011). Our results show reductions in repetitive behaviors and hyperactivity and are in accordance with risperidone’s efficacy in a genetic knock-out mouse model of ASD using a somewhat higher intraperitoneal dose of 0.2 mg/kg (Penagarikano et al. 2011). In a study by Gould et al. (2011), risperidone at the dose of 0.1 mg/kg also reduced marble burying, another form of repetitive behavior in an idiopathic ASD mouse model. The significant reduction in repetitive behaviors achieved by all 3 compounds including circling and excessive grooming as well as hyperactivity may be linked to their actions at dopamine D_2_ receptors (Hertel et al. 1996; Kikuchi et al. 1995; Kiss et al. 2010). There is also evidence that the serotonin 5-HT_2A_ receptor antagonist M100907 improved cognitive inflexibility, a form of higher order repetitive behavior in BTBR mice (Amodeo et al. 2014), which implies that antagonism at the 5-HT_2A_ receptor may equally play a role in the antirepetitive efficacy of all the 3 investigated compounds (Gyertyán et al. 2011).

In summary, cariprazine, an atypical antipsychotic with a unique profile of receptor affinities and mechanism of actions, was effective on multiple behavioral endpoints of the prenatal VPA-exposure model. The differentiated efficacy of cariprazine on social readouts may forecast a therapeutic benefit for the core symptoms of ASD. While acknowledging the limitations of any preclinical animal model and the lack of further supportive results obtained from other ASD models, future clinical studies in human subjects are warranted to extend our knowledge on the possible effectiveness of cariprazine in the ASD population.
